# Fatal Rocky Mountain Spotted Fever along the United States–Mexico Border, 2013–2016

**DOI:** 10.3201/eid2310.170309

**Published:** 2017-10

**Authors:** Naomi A. Drexler, Hayley Yaglom, Mariana Casal, Maria Fierro, Paula Kriner, Brian Murphy, Anne Kjemtrup, Christopher D. Paddock

**Affiliations:** Centers for Disease Control and Prevention, Atlanta, Georgia, USA (N.A. Drexler, C.D. Paddock);; Arizona Department of Health Services, Phoenix, Arizona, USA (H. Yaglom, M. Casal);; Imperial County Public Health Department, El Centro, California, USA (M. Fierro, P. Kriner);; County of San Diego Health and Human Services Agency, San Diego, California, USA (B. Murphy);; California Department of Public Health, Sacramento, California, USA (A. Kjemtrup)

**Keywords:** Rocky Mountain spotted fever, US–Mexico border, rickettsia, tickborne disease, vectorborne infections, vector-borne infections, California, Arizona, United States, Mexico, Rickettsia rickettsii

## Abstract

Rocky Mountain spotted fever (RMSF) is an emerging public health concern near the US–Mexico border, where it has resulted in thousands of cases and hundreds of deaths in the past decade. We identified 4 patients who had acquired RMSF in northern Mexico and subsequently died at US healthcare facilities. Two patients sought care in Mexico before being admitted to US-based hospitals. All patients initially had several nonspecific signs and symptoms, including fever, headache, nausea, vomiting, or myalgia, but deteriorated rapidly without receipt of a tetracycline-class antimicrobial drug. Each patient experienced respiratory failure late in illness. Although transborder cases are not common, early recognition and prompt initiation of appropriate treatment are vital for averting severe illness and death. Clinicians on both sides of the US–Mexico border should consider a diagnosis of RMSF for patients with rapidly progressing febrile illness and recent exposure in northern Mexico.

## Abstract 2

Although these cases are uncommon, early recognition and prompt initiation of appropriate treatment are vital for averting severe illness and death.

Rocky Mountain spotted fever (RMSF), a life-threatening and rapidly progressing tickborne disease, is caused by infection with the bacterium *Rickettsia rickettsii*. Onset of infection is characterized by nonspecific signs and symptoms that include fever, headache, and muscle pain. Progressing damage to the vascular endothelium can result in organ failure, cutaneous necrosis, and death. RMSF is frequently fatal for persons who do not receive appropriate therapy with a tetracycline-class drug during the first 5 days of illness; half of all deaths occur within the first 8 days (*1*).

In the United States, RMSF is characteristically a rare and sporadically distributed disease: most cases are reported from mid-Atlantic states (*2*). Recently, however, epidemic levels of RMSF have been described for areas of eastern and southern Arizona and northern Mexico (*3*–*6*). Transmission in these areas is perpetuated by large numbers of brown dog ticks (*Rhipicephalus sanguineus* sensu lato), which are responsible for unusually high incidence of disease in this region (*3*,*5*,*7*). *Rhipicephalus *tick–transmitted RMSF was initially recognized in Mexico during the 1940s, yet during the past 12 years the disease has rapidly reemerged in parts of Baja California and Sonora, Mexico (*3*,*4*,*6*,*8*,*9*). We describe 4 patients who acquired RMSF in Mexico and subsequently sought care in the United States. These cases highlight the need for increased healthcare provider awareness of this rapidly progressing disease in communities on both sides of the border.

## Methods

During 2013–2016, the Arizona Department of Health Services, the California Department of Public Health (CDPH), and the US Centers for Disease Control and Prevention (CDC) identified 4 cases of RMSF in persons who acquired the illness in Mexico and later died in the United States (Table). The cases were identified during the course of routine surveillance and diagnostic testing for this disease at the respective state public health laboratories or CDC. To better characterize the epidemiologic risk factors, clinical progression, and treatment course associated with each of these deaths, we performed a retrospective review of clinical and epidemiologic data and, when available, medical charts. Because the CDC Human Subjects Review Committee determined that this evaluation was not research, this case series was exempt from institutional review board and human subjects review.

**Table:** Selected epidemiologic and clinical elements from patients with fatal cases of RMSF along the US–Mexico border, 2013–2016*

Element	Case-patient 1	Case-patient 2	Case-patient 3	Case-patient 4
Patient history				
Known exposure in RMSF-epidemic area of Mexico	+		+	+
Evidence of receipt of medical care in Mexico	+			+
Prescribed nontetracycline antimicrobial drug	+	+	+	+
Signs and symptoms at initial presentation				
Fever	+	+	+	+
Headache		+		+
Nausea/vomiting/diarrhea		+	+	
Rash				
Severe end-stage manifestations				
Skin necrosis	+	+		+
Rash	+	+	+	+
Respiratory failure	+	+	+	+
Disseminated intravascular coagulation		+		

*RMSF, Rocky Mountain spotted fever; +, present; blank cells, absent.

## Case Reports

### Case 1

In December 2013, fever, chills, and thrombocytopenia developed in a 22-year-old man while he was attending school in Hermosillo, Mexico. While visiting family, he sought care at a hospital in Nogales, Mexico, where he received treatment with penicillin and was released. He subsequently sought care at an emergency department in Nogales, Arizona, USA, where he was found to have fever; hypotension; hepatomegaly; splenomegaly; thrombocytopenia; and elevated levels of creatinine, hepatic transaminases, and bilirubin. He was given intravenous vancomycin and piperacillin/tazobactam and transferred to a tertiary care facility in Tucson, Arizona. Admitting documents at the tertiary care facility noted acute kidney and liver failure. He later became acidotic; his altered mental status and respiratory failure progressed, and he was intubated. Subsequently, the patient experienced a dusky and violaceous rash, bilateral necrosis of his hands and feet, followed by gangrene and severe edema. He remained in an intensive care unit for 2 weeks before experiencing cardiac arrest; he died ≈3 weeks after symptom onset. 

Testing of serum obtained on day 19 of the patient’s illness revealed reciprocal IgM and IgG titers reactive with *R. rickettsii* of 1,024 each, according to an indirect immunofluorescence antibody assay performed at a commercial laboratory. A skin punch biopsy specimen obtained from a rash lesion on the lower abdomen ≈2 weeks after illness onset revealed spotted fever group *Rickettsia* (SFGR) antigens in the endothelial cells of inflamed small blood vessels in the dermis. The sample was tested by an immunohistochemical stain for SFGR at CDC (*10*) (Figure, panel A).

**Figure:**
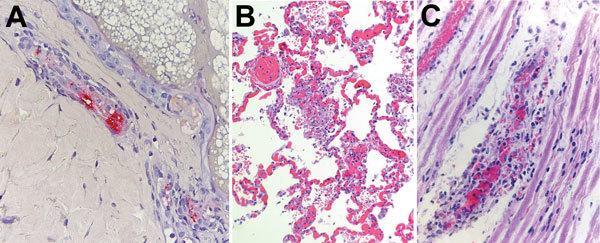
Histologic slides of autopsy tissue from patients who acquired Rocky Mountain spotted fever in northern Mexico and died at hospitals in the United States, 2013–2016. A) Immunohistochemical stain of *Rickettsia rickettsii* antigens (red) in inflamed blood vessel adjacent to eccrine gland in a skin biopsy specimen from case-patient 1. Immunoalkaline phosphatase with naphthol-fast red and hematoxylin counterstain; original magnification ×50. B) Diffuse pulmonary capillaritis in case-patient 4. Hematoxylin and eosin stain; original magnification ×50. C) Vasculitis involving a small blood vessel in a peripheral nerve of case-patient 4. Hematoxylin and eosin stain; original magnification ×100.

### Case 2

In May 2014, a 52-year-old woman from Calexico, California, USA, sought care at an emergency department in El Centro, California, after 3 days of fever, diarrhea, nausea, and vomiting. For the previous 4 days, the patient had self-medicated with ampicillin obtained in Mexico for a toothache. She was hospitalized and received intravenous levofloxacin and cefepime for presumed urosepsis; on day 3 of hospitalization (day 6 of illness), she was transferred to a tertiary care facility in San Diego, California. At arrival, scattered petechiae were visible on her upper and lower extremities; the medical chart reported disseminated intravascular coagulation (DIC) with pancytopenia. Laboratory results showed increased clotting time (elevated international normalized ratio) and elevated levels of hepatic transaminases, but no D-dimers or fibrogen levels were reported. The patient was intubated and subsequently experienced encephalopathy, cardiomyopathy, and acute renal failure requiring hemodialysis. Intravenous vancomycin and metronidazole were started. The patient never received a tetracycline-class antimicrobial drug before dying of complications of DIC on day 28. 

An autopsy revealed bilateral pyelonephritis, acute pancreatitis, pneumonia, ascites, extensive cutaneous necrosis, and widespread ischemic damage. A serum specimen obtained on day 7 of illness revealed a reciprocal IgG titer of <64 and a reciprocal IgM titer of 160, reactive with *R. rickettsii* when tested by an immunofluorescence antibody assay at CDPH. A serum sample obtained on day 24 showed reciprocal IgG and IgM titers of >1,024 and >160, respectively. A skin biopsy specimen of the rash lesion obtained after death and tested by PCR at CDPH was positive for DNA of SFGR species. 

The patient had not reported travel for the 1 month preceding illness onset, but relatives frequently visited family in Mexicali, Mexico, and brought their pet dogs across the border with them. An ecologic assessment of the patient’s home in Calexico revealed an extensive brown dog tick infestation of the dogs and the yard. A total of 37 ticks were collected from the domestic and peridomestic setting and tested by PCR at CDPH. One of the 37 ticks was positive for DNA of a *Rickettsia* species. Subsequent testing of this specimen at CDC by a genotyping assay for this agent led to identification of *R. rickettsii* (*11*).

### Case 3

On 2 occasions in September 2014, a 39-year-old man sought care at a healthcare facility in Riverside County, California, for fever, cough, dyspnea, diarrhea, nausea, vomiting, and abdominal pain. Both times he was sent home with a suspected diagnosis of viral syndrome. His condition worsened, and he sought care at a third facility on day 3 of his illness, at which time leukopenia and thrombocytopenia were reported, and a chest radiograph showed pulmonary infiltrates suggestive of pneumonia; a mottled rash also appeared on his extremities. He was hospitalized and given vancomycin, imipenem, azithromycin, and metronidazole. Subsequently, he experienced respiratory failure, requiring ventilator assistance. On day 7, he was given doxycycline, and admitted to an intensive care unit. On day 16, he died. 

A plasma specimen obtained on day 7 revealed DNA of an SFGR species when tested by a real-time PCR at CDC (*12*). No autopsy was performed. The patient had frequently traveled to Mexicali; his most recent trip was 1 week before illness onset.

### Case 4

On 3 occasions in March 2016, an 18-year-old woman sought care in Nogales, Mexico, for fever, headache, myalgia, fatigue, and arthralgia. After each visit she was sent home with palliative treatment for fever, and after 1 of the visits, cephalexin was prescribed for an unspecified illness. On day 7 of illness, she sought care at an emergency department in Nogales, Mexico, for abdominal pain, rash, headache, and extreme fatigue. Laboratory testing detected leukocytosis, thrombocytopenia, and elevated levels of pancreatic enzymes and hepatic transaminases. The patient was transported across the border to Nogales, Arizona, for further medical care, but she died of cardiac arrest at arrival. 

An autopsy revealed a widespread petechial rash; perivascular inflammation of the heart, lungs, and liver; and petechial hemorrhages in the epicardium and lung pleura. Postmortem specimens of whole blood, urine, and vitreous humor were positive for DNA of *R. rickettsii* when tested by PCR at CDC; immunohistochemical assay, also performed at CDC, demonstrated abundant intravascular antigens of SFGR in sections of lung, liver, heart, spleen, and central nervous system tissue (Figure, panels B and C).

## Discussion

During 2013–2016, passive surveillance identified 4 cases of fatal RMSF in persons who had traveled to or resided in areas of northern Mexico and who died in the United States. Epidemic RMSF is an emerging public health concern in portions of northern Mexico (*3*,*4*,*13*). During 2009–2016, a total of 967 cases of RMSF, including 132 deaths, were reported in Mexicali (*3*). Similarly, during 2004–2015, a total of 1,129 cases and 188 deaths from RMSF were reported from Sonora, Mexico, prompting the Secretary of Health in Mexico to declare an epidemiologic emergency (*13*). Cases of RMSF have also been increasingly reported from the Mexico states of Coahuila and Chihuahua. During 2015, approximately 181,300,000 persons crossed into the United States from a Mexico land border (23,800,000 into Arizona, 72,400,000 into California, 2,400,000 into New Mexico, and 82,700,000 into Texas), making these border crossings some of the busiest in the world (*14*). Although transborder cases of RMSF could be infrequent, they underline the need for improved clinical awareness regarding the diagnosis and treatment of this disease on both sides of the border, and they highlight the value of ongoing communication between health authorities in the United States and Mexico.

Each of the 4 patients we report sought care at a healthcare facility for fever and other nonspecific signs and symptoms including headache, nausea, vomiting, or myalgia; thrombocytopenia was reported during early illness (within the first 3–4 days of illness) for at least 2 patients. Rash was observed for all patients during the course of their illness but was not noted in the original clinical description for any. Two patients sought care in Mexico before being admitted to US-based facilities, and all patients reported having made multiple visits to healthcare facilities before admission. All patients sought care within the first 3 days of illness and were admitted to the hospital within 7 days of illness onset, reflecting the rapidly progressing nature of RMSF. Respiratory failure and cutaneous necrosis of the extremities were common end-stage manifestations of this severe disease. DIC was a reported end-stage manifestation for 1 patient but was not validated by specific laboratory assays. True DIC is rarely documented in cases of RMSF (*15*). Among the 4 patients, death occurred 7–28 days after illness onset, and several patients received life support before death. Each case met >1 laboratory criteria for a confirmed spotted fever rickettsiosis (*16*).

Early suspicion of RMSF and prompt initiation of tetracycline-class antimicrobial drug therapy are critical for averting severe sequelae and death from RMSF (*17*,*18*). In this case series, all patients received a non–tetracycline-class antimicrobial drug within the first week of illness, and only 1 patient received doxycycline at any point during illness. Doxycycline should be initiated immediately whenever a rickettsial disease, including RMSF, is suspected and should never be delayed while awaiting the appearance of a rash or confirmatory laboratory result. Clinicians along the US–Mexico border should be cognizant of the occurrence of RMSF in this region and should consider this diagnosis for patients with otherwise unexplained febrile or septic syndromes. RMSF can be difficult to distinguish from various other life-threatening infectious and noninfectious conditions, including measles, leptospirosis, thrombotic thrombocytopenic purpura, and meningococcemia, particularly during the early stages of disease (*19*). Differentiation becomes even more challenging in light of recent epidemics of arboviral infections such as those caused by Zika, dengue, and chikungunya viruses, which can have similar signs and symptoms early in illness, including fever, myalgia, arthralgia, and rash (*20*,*21*). Results of blood chemistries, including complete blood counts and hepatic function panels, may help distinguish between these infections. For example, platelet levels <100,000 cells/mm^3^ are rarely found in patients with Zika or chikungunya virus infection but are often found in patients with advanced RMSF and dengue, particularly those with dengue hemorrhagic fever (*19*–*21*). Lymphopenia is often found in patients infected with chikungunya virus but less commonly found in patients infected with Zika and dengue viruses (*20*,*21*). Leukocyte counts are typically within reference range or slightly elevated in patients with RMSF, although patients with more advanced stages of disease may have lymphocytosis with a predominant left shift (*4*,*7*,*19*).

Questions about epidemiologic risk factors are helpful for clinical evaluations. Of the 4 patients reported here, 3 had spent time in regions of northern Mexico recognized for high rates of RMSF (*3*). At least 1 patient reported having been bitten by a tick in the week before illness onset, and 2 reported having had contact with dogs. 

Reduction of duplication and delayed access to lifesaving care for patients with RMSF can be facilitated by increased clinical awareness, in-depth clinical and social histories, and improved binational communication. Increased clinical education along the US–Mexico border can help clinicians correctly recognize and promptly treat suspected cases of RMSF.
